# Tricuspid valve metastasis and tumoural pulmonary embolism case report: a rare germ cell tumour manifestation

**DOI:** 10.1093/ehjcr/ytag594

**Published:** 2026-08-08

**Authors:** Julio Ernesto Peralta-Rodríguez, Carlos A Jimenez-Cabezudo, José Paúl Huaccha-Rodríguez, Danny R Sánchez-Galarza

**Affiliations:** Cardiovascular and Thoracic Surgery Department, Hospital Nacional Dos de Mayo, Av. Miguel Grau cdra. 13, Cercado de Lima, Lima 15003, Peru; Cardiovascular and Thoracic Surgery Department, Hospital Nacional Dos de Mayo, Av. Miguel Grau cdra. 13, Cercado de Lima, Lima 15003, Peru; Cardiovascular and Thoracic Surgery Department, Hospital Nacional Dos de Mayo, Av. Miguel Grau cdra. 13, Cercado de Lima, Lima 15003, Peru; Cardiovascular and Thoracic Surgery Department, Hospital Nacional Dos de Mayo, Av. Miguel Grau cdra. 13, Cercado de Lima, Lima 15003, Peru

**Keywords:** Cardiac tumour, Tricuspid metastases, Germ cells, Cardiac metastases, Case report

## Abstract

**Background:**

Cardiac metastases are a rare complication, with an incidence ranging from 2.3% to 18.3%. While secondary heart neoplasms are more common than primary ones, germ cell tumour (GCT) involvement is rare, with less than 30 cases reported globally. Mixed GCTs of gonadal location are a type of cancer that rarely metastasize the heart.

**Case summary:**

We present the case of a 31-year-old male with a history of testicular GCT. A few months after radical right orchiectomy, he developed respiratory distress and heart failure, associated with the presence of masses in the tricuspid valve and pulmonary branches. He was scheduled for tricuspid valve replacement surgery and removal of pulmonary masses. Histopathology determined a GCT: teratoma with immature and mature elements.

**Discussion:**

Metastatic lesions from GCTs can clinically mimic infective endocarditis or pulmonary embolism, potentially leading to diagnostic delays in oncological patients. The case highlights the importance of considering metastatic aetiology in cancer patients with cardiac masses for timely surgical intervention.

Learning points

- Metastatic lesions from germ cell tumours can clinically mimic infective endocarditis or pulmonary embolism, potentially causing severe diagnostic delays.
- Intracardiac metastasis requires high suspicion in patients with right heart strain, where surgical excision and valve replacement offer effective salvage.

## Introduction

Germ cell tumours (GCTs) are rare neoplasms arising from primordial germ cells. Nevertheless, in young men, malignant GCTs are the most common solid malignancy, with an incidence of 7–8 per 100 000 population.^1^

Cardiac metastasis from mixed GCTs of gonadal origin is exceptionally rare. When present, it typically results from direct invasion of adjacent organs such as the lungs or mediastinal lymph nodes, or from haematogenous spread through the gonadal veins.^2^ Clinical manifestations generally depend on the anatomical location of the metastasis. Haematogenous dissemination predominantly affects the right chambers of the heart via the inferior vena cava and may lead to right-sided heart failure.^2^

We report the case of a 31-year-old man admitted to our hospital. Three months earlier, he underwent a radical right orchiectomy for a primary non-seminomatous testicular GCT. Subsequently, he presented with progressive respiratory distress mimicking pulmonary thromboembolism.

## Summary figure

**Figure:**
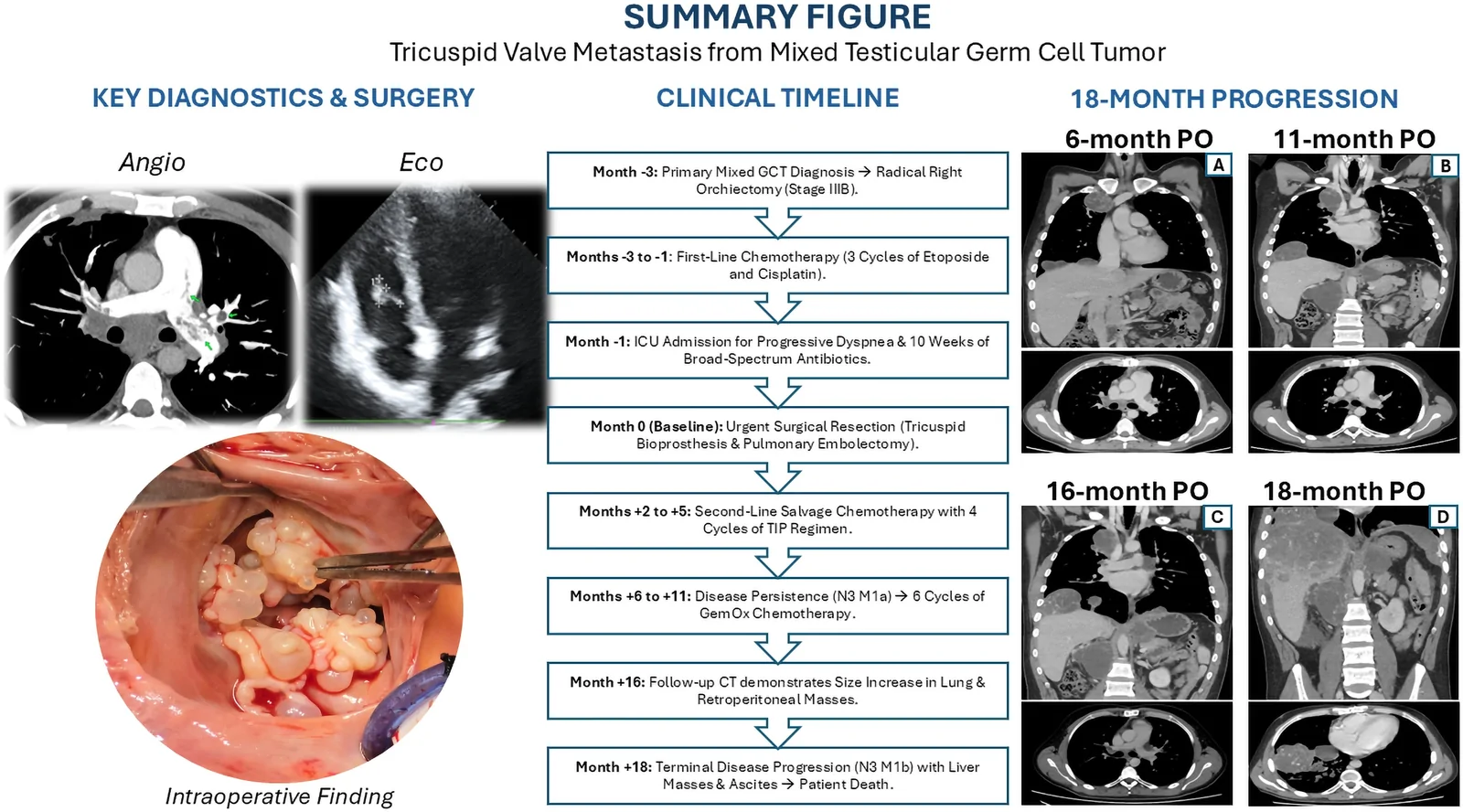
Multimodality educational roadmap of a tricuspid valve metastasis and tumoural pulmonary embolism. The central abstract illustrates the diagnostic pathway and treatment course across a standardized chronological timeline where Month 0 represents the cardiac intervention. Left panel shows the pre-operative TTE and angiogram detailing tricuspid valve and pulmonary artery masses, alongside the definitive macroscopic operative view of clustered vesicular teratoma lesions infiltrating the tricuspid valve. Central panel maps out the precise chronological progression from negative timeline milestones (primary orchiectomy and first-line chemotherapy at Months −3 to −1) through baseline surgical clearance (Month 0) and subsequent post-operative salvage regimens (TIP and GemOx from Months +2 to +11). Right panel provides the long-term surveillance computed tomography (CT) continuum, tracing initial post-surgical stability up to terminal locoregional, hepatic, and pulmonary visceral progression at Month +18 (Stage N3 M1b).

## Case presentation

A 31-year-old male with a 3-month history of a primary GCT diagnosed at the National Institute of Neoplastic Diseases, presenting with CT-documented pulmonary involvement (clinical stage IIIB), underwent a radical right orchiectomy. Histopathological evaluation revealed a mixed GCT comprising classic seminoma, embryonal carcinoma, yolk sac tumour, and postpubertal teratoma. Following surgery, the patient received three cycles of chemotherapy with etoposide and cisplatin.

He subsequently developed progressive dyspnoea and required transfer to the ICU for mechanical ventilation and broad-spectrum antibiotics (meropenem and vancomycin) due to suspected hospital-acquired pneumonia. Transthoracic echocardiography (TTE) revealed masses suggestive of vegetations involving the tricuspid valve, subvalvular apparatus, and pulmonary valve. Blood and urine cultures were negative. Due to suspicion of pulmonary thromboembolism and tricuspid endocarditis, a CT pulmonary angiogram was performed, showing signs of chronic thromboembolism at the pulmonary artery bifurcation. After completing 10 weeks of antibiotics, the patient was referred to our centre for surgical evaluation.

**Figure 1:**
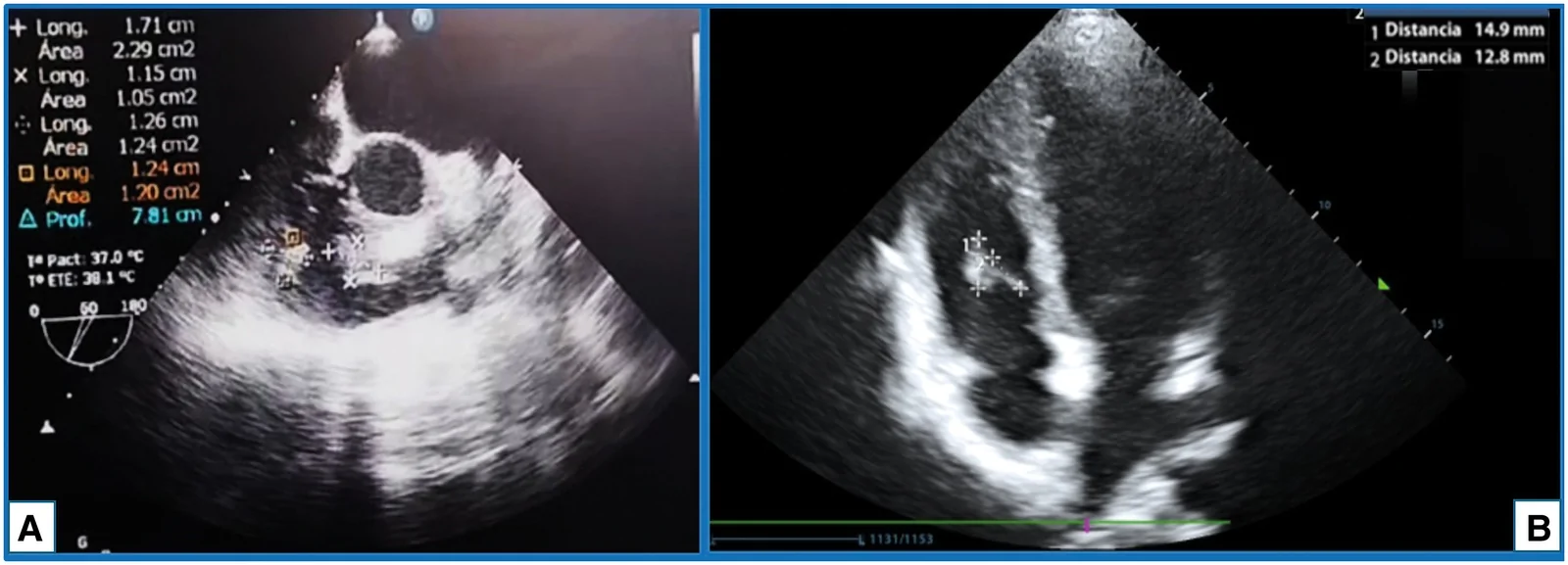
Pre-operative echocardiographic evaluation of the intracardiac metastatic masses. (*A*) Transoesophageal echocardiogram demonstrating multiple heterogeneous, lobulated tumour masses within the right ventricular outflow tract and tracking towards the pulmonary artery trunk. (*B*) Transthoracic echocardiogram apical four-chamber view showing two distinct, mobile, heterogeneous masses firmly attached to the tricuspid valve and subvalvular apparatus.

**Figure 2:**
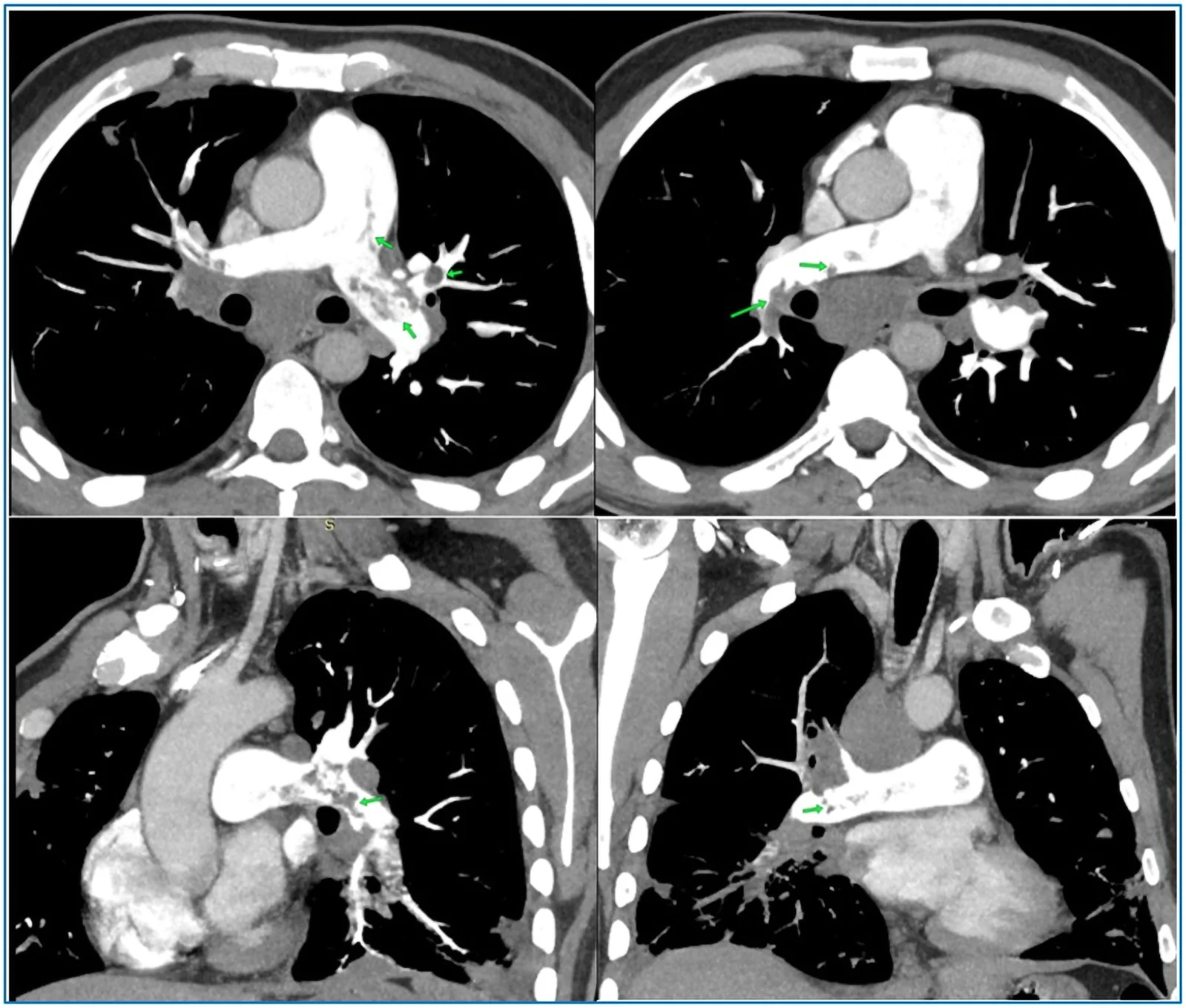
Computed tomography pulmonary angiogram. Views demonstrating filling defects and signs of chronic pulmonary thromboembolism at the main pulmonary artery bifurcation and extending into the lobar branches.

On admission, he was afebrile, haemodynamically stable, breathing spontaneously, and no longer on antibiotics. Electrocardiogram showed normal sinus rhythm. Transthoracic echocardiography showed preserved biventricular function and two heterogeneous masses attached to the tricuspid subvalvular apparatus (1.5 × 0.9 cm and 1.2 × 0.6 cm), as well as a mobile heterogeneous mass (1.5 × 1 cm) protruding through the pulmonary valve (*Figure 1B*). Transoesophageal echocardiography identified multiple masses in the right ventricular outflow tract (RVOT) and pulmonary artery (*Figure 1A*). Computed tomography images showed chronic pulmonary thromboembolism in the main and lobar pulmonary arteries (*Figure 2*).

Laboratory tests revealed severe leucopenia and neutropenia; C-reactive protein was normal, and blood cultures remained negative. The patient received filgrastim until normal leucocyte counts were achieved and was scheduled for surgery.

**Figure 3:**
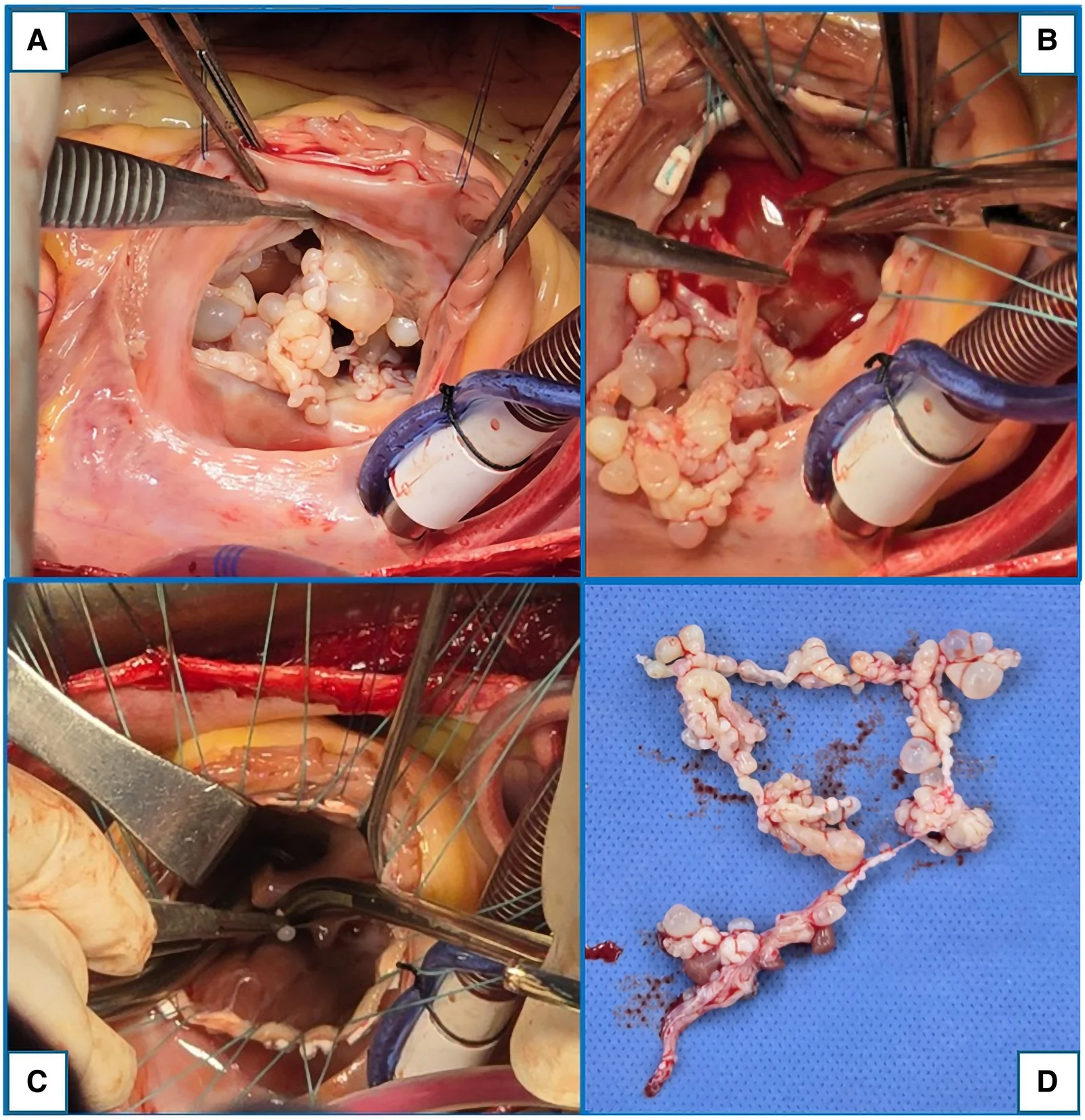
Intra-operative findings and surgical resection of the intracardiac metastasis. (*A*) Right atriotomy exposure demonstrating a severely degenerated tricuspid valve completely infiltrated by multiple, highly mobile, clustered vesicular tumour lesions. (*B*) En bloc surgical excision of the tricuspid valve leaflets along with the adherent subvalvular tumour masses. (*C*) Structural preparation of the tricuspid annulus with pledgeted mattress sutures positioned for the subsequent implantation of the bioprosthetic valve. (*D*) Gross macroscopic appearance of the completely resected, multilocular, vesicular tumour specimens tracking from the right ventricle to the pulmonary circulation, which histopathology confirmed as mature and immature teratoma.

**Figure 4:**
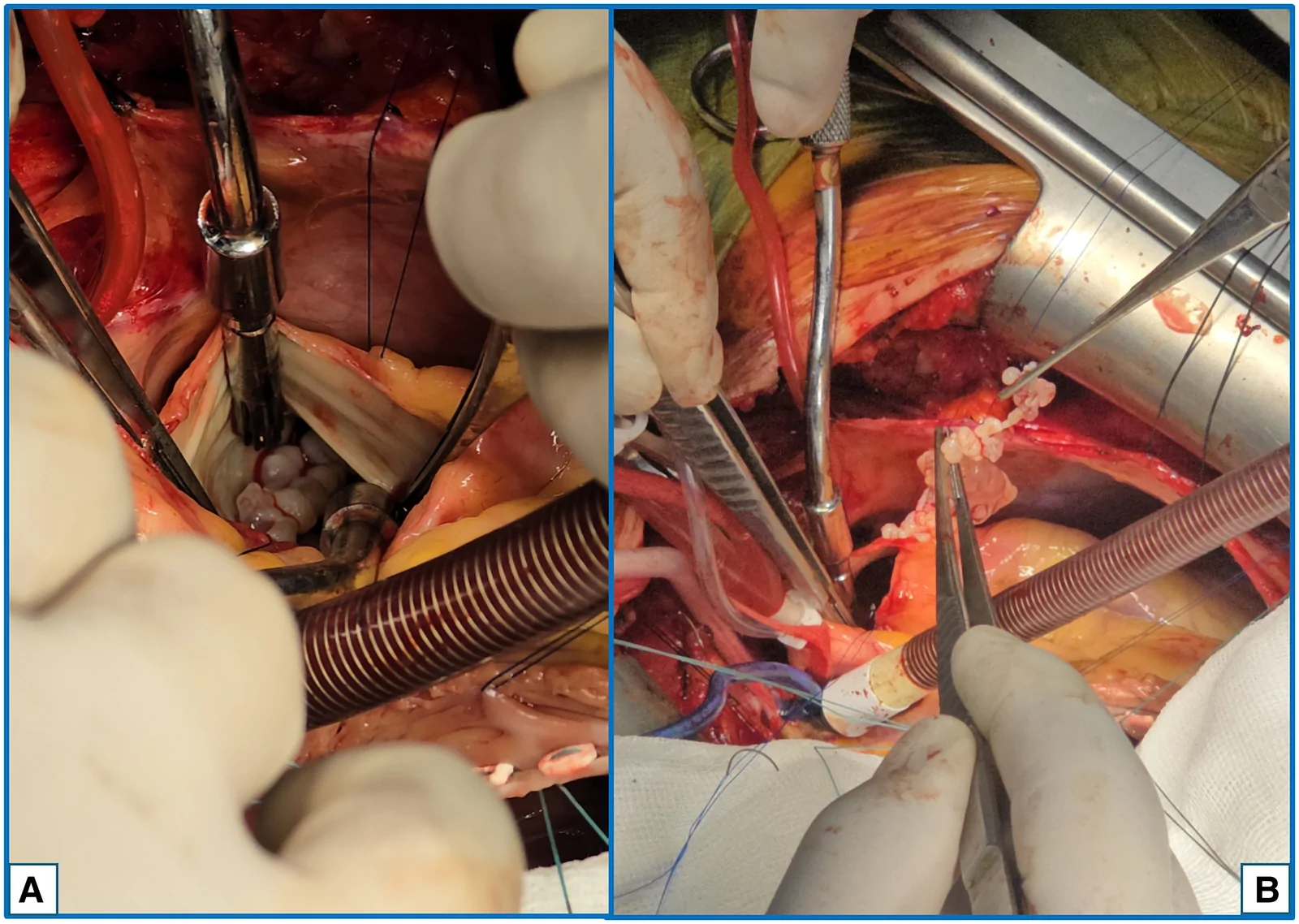
Pulmonary arteriotomy and resection of tumoural emboli. (*A*) Direct exposure of the main pulmonary artery trunk through a longitudinal arteriotomy, revealing multiple white-to-translucent vesicular tumour masses occluding its lumen and right pulmonary artery origin. (*B*) Surgical extraction and clearance of the chemoresistant teratomatous fragments from the pulmonary circulation using vascular forceps, confirming gross tumoural embolization downstream from the right ventricular outflow tract.

We performed a standard cardiopulmonary bypass with aortic and bicaval cannulation. Cardiac arrest was achieved with intermittent antegrade cardioplegia. Through right atriotomy, a degenerative tricuspid valve with multiple vesicular lesions extending to the RVOT was identified (*Figure 3A*). En bloc excision of the tricuspid valve and tumour mass was performed (*Figure 3B*). Due to suspicion of residual tumour, the pulmonary artery trunk was incised, allowing extraction of remaining lesions from the pulmonary trunk and right pulmonary artery (*Figure 4*). Frozen-section biopsy revealed a mature cystic teratoma.

A comprehensive exploration of the right ventricle, RVOT, PAT, and RPA confirmed the absence of remaining tumour. A bioprosthetic valve size 29 was implanted in tricuspid position. The patient was weaned from bypass without complications and transferred to recovery, haemodynamically stable and extubated.

Final histopathology confirmed a GCT: teratoma with immature and mature components. Post-operative TTE showed preserved ventricular function and a competent tricuspid prosthesis with no residual tumour. The post-operative course was uneventful, and the patient was discharged 10 days later.

**Figure 5:**
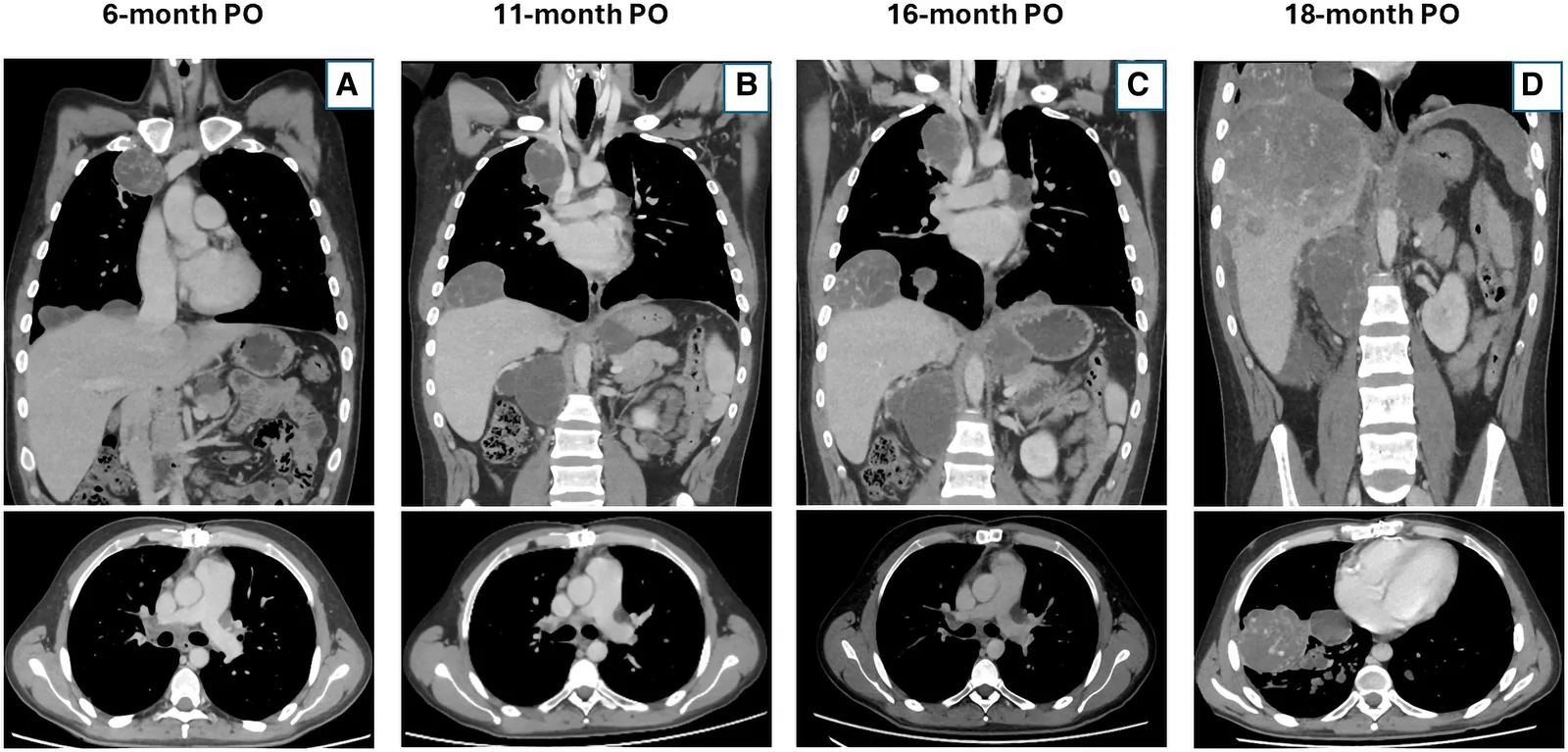
Serial post-operative chest and abdominal computed tomography scans demonstrating disease progression. (*A*) At 6 months, (*B*) 11 months, and (*C*) 16 months post-op (PO), coronal (top) and axial (bottom) views show stable post-surgical findings with no evidence of intracardiac recurrence. (*D*) At 18 months post-op, scans reveal a significant locoregional and distant disease progression, characterized by a large, heterogeneous, lobulated mass in the right lung base (arrow) accompanied by extensive hepatic and retroperitoneal metastatic lesions.

Following hospital discharge, the patient continued outpatient oncological follow-up. Two months after cardiac surgery, he initiated salvage chemotherapy with the TIP regimen (paclitaxel, ifosfamide, and cisplatin), successfully completing four cycles. Throughout this period, the patient remained asymptomatic, maintaining an ECOG performance status of 1. After completing the four chemotherapy cycles, a contrast-enhanced CT scan of the chest, abdomen, and pelvis was performed, revealing multiple masses measuring approximately 60 × 56 mm in right lung, alongside retrocaval, right renal hilar, and retroperitoneal lymphadenopathies (staged as N3 M1a). No lesions suggestive of tumour recurrence within the mediastinal vascular structures were visualized (*Figure 5A*).

Due to disease persistence, a second-line salvage chemotherapy regimen with gemcitabine and oxaliplatin (GemOx) was initiated. The patient received six cycles of this regimen over 5 months. A subsequent follow-up CT scan showed no significant changes in the masses, nodules, or adenopathies compared to the previous study, with persistent retroperitoneal lymph node conglomerates (N3 M1a) (*Figure 5B*). The patient remained asymptomatic (ECOG 1) and continued under oncological surveillance.

At 16 months post-op, a follow-up CT scan demonstrated a size increase in the right lung masses and nodular lesions, as well as progressive retroperitoneal masses (*Figure 5C*). At 18 months post-op, another CT scan was performed due to worsening dyspnoea and jaundice; the imaging revealed a heterogeneous liver with a mass in the right hepatic lobe causing biliary tract compression, a further increase in the size of the retroperitoneal and coeliac trunk masses, and abundant free peritoneal fluid (ascites), representing terminal disease progression (N3 M1b) (*Figure 5D*; see also Summary figure). Subsequently, the patient experienced severe clinical deterioration and passed away 18 months after the cardiac surgery.

## Discussion

Fewer than 30 cases of intracardiac metastasis from GCTs via haematogenous dissemination have been reported worldwide, making this the first documented case in our country. Primary cardiac tumours are extremely rare, with an autopsy incidence of 0.001%–0.3%. Approximately 75% are benign—most commonly atrial myxoma—while malignant tumours such as rhabdomyosarcoma and angiosarcoma represent the minority.^3^ Secondary cardiac tumours are far more common. Autopsy studies show cardiac metastatic involvement in 2.3%–18.3% of cancer patients, most frequently from melanoma, lung carcinoma, breast carcinoma, and oesophageal cancer; GCTs represent an exceptional aetiology.^3^

A 35-year autopsy study demonstrated that pericardial involvement is the most frequent metastatic pattern (57.3%), followed by myocardial infiltration; endocardial metastasis is the least common.^3,4^

In a review of 17 published cases of GCTs with cardiac metastases, 16 patients were men with seminoma or non-seminomatous tumours. In 11 cases, metastases were in the right heart chambers—consistent with our case—four involved the left chambers and two involved the pericardium.^5^

In our institution, this represents the 11th case of intracardiac mass identified this year, the second of malignant origin, and the first of metastatic aetiology.

Cardiac metastases often occur late in the course of malignancy and produce clinical symptoms in only about 10% of affected patients. Symptoms are typically determined by the anatomical location of the tumour rather than tumour histology. Although many patients remain asymptomatic, cardiac tamponade—one of the earliest and most common manifestations—may occur. Malignant cardiac involvement may also cause arrhythmias or heart failure. In right-sided involvement, previously reported symptoms include right heart failure, dyspnoea, and vena cava thrombosis. Tumoural pulmonary embolism, although rare, has also been described and occurred in this patient. In this case, embolic tumour masses were surgically extracted directly through pulmonary arteriotomy, with favourable findings on post-operative imaging.^5,6^

Right-sided intracardiac metastases frequently pose an extraordinary diagnostic dilemma; in our patient, their high mobility and vesicular margins closely mimicked infective endocarditis vegetations, leading to a 10-week management delay. This infectious diagnosis was definitively ruled out due to persistently sterile cultures, normal inflammatory markers, and lack of clinical response to broad-spectrum antibiotics. In non-seminomatous GCTs, this presentation is highly illustrative of the growing teratoma syndrome (GTS), as confirmed by our patient’s frozen-section and final biopsy, which revealed exclusively mature and immature teratoma components. While first-line cisplatin-based chemotherapy successfully eradicated his initial aggressive malignant elements, it induced a selection pressure that spared these chemoresistant teratoma cells. These residual clones subsequently migrated and proliferated paradoxically within the tricuspid subvalvular apparatus and pulmonary circulation. Because GTS is entirely unresponsive to cytotoxic chemotherapy, urgent radical surgical excision remains the absolute therapeutic pillar to avert imminent mechanical outflow tract obstruction, offering favourable short-term outcomes despite late systemic progression.^2,7,8^

Long-term prognosis of testicular GCT metastasis to the heart depends on histologic subtype and completeness of surgical resection.^9,10^ In most reported cases of right-sided intracardiac metastasis, complete surgical excision combined with adjuvant chemotherapy has been associated with favourable short-term outcomes.^5^ Thorough evaluations of tumour extent are essential in determining operability. Echocardiography serves as a useful initial diagnostic and follow-up tool; however, imaging modalities such as CT or MRI are required to accurately assess tumour extension, which ultimately determines prognosis.^11^

We present an uncommon case involving a 31-year-old man with a history of radical right orchiectomy for a gonadal GCT, who subsequently developed cardiac metastasis involving the tricuspid valve, right ventricle, and tumoural embolic masses within the pulmonary artery following chemotherapy. The initial clinical impression at his referring hospital was infectious endocarditis; however, although this is an infrequent complication, it is essential to consider this pattern of dissemination in patients with this type of tumour. Early recognition and timely surgical management significantly improve the prognosis after resection of metastatic cardiac masses.

## Data Availability

The data supporting the findings of this case report are documented within the patient’s medical record at our institution and are available from the corresponding author upon reasonable request, subject to compliance with patient confidentiality and institutional privacy policies.

## Lead author biography

Julio Ernesto Peralta-Rodríguez is a thoracic and cardiovascular surgeon. He currently is the Chief Surgeon of Thoracic and Cardiovascular Surgery Department at the Hospital Nacional Dos de Mayo in Lima (Peru), a national reference centre for complex cardiac care. He graduated from San Marcos National University and has extensive experience in managing adult and congenital heart diseases. He commands many medical missions, an initiative dedicated to decentralize cardiac surgery and expand access to life-saving interventions around Peru. His clinical and research interests focus on improving surgical outcomes in valvular pathologies, cardiac congenital diseases, and enhancing public health strategies for cardiovascular disease prevention.

## Notes

**Consent:** Written informed consent was obtained from the patient for publication of this case report and any accompanying images. A copy of the written consent is available for review by the editor-in-chief of this journal.
